# (1S*,2R*,3S*,4R*,5R*)-5-Tetra­decyloxy­methyl-7-oxabicyclo­[2.2.1]heptane-2,3-dicarb­oxy­lic anhydride

**DOI:** 10.1107/S1600536812046259

**Published:** 2012-11-17

**Authors:** Colin N. Kelly, Sarah M. Sulon, Lam N. Pham, Kang Rui Xiang, Richard E. Sykora, David C. Forbes

**Affiliations:** a Department of Chemistry and Biochemistry, Oberlin College, Oberlin, OH 44074, USA; b Biology Department, Elizabethtown College, Elizabethtown, PA 17022, USA; c Department of Chemistry, University of South Alabama, Mobile, AL 36688, USA

## Abstract

In the title compound, C_23_H_38_O_5_, the oxabicyclo­[2.2.1]heptane-2,3-dicarb­oxy­lic anhydride unit has a normal geometry and the tetra­decoxymethyl side chain is fully extended. In the crystal, mol­ecules are linked head-to-head by C—H⋯O hydrogen bonds, forming two-dimensional networks propagating along the *a* and *c*-axis directions.

## Related literature

Olefinic hydrogenation of an oxabicyclo­[2.2.1]hept-5-ene derivative using catalytic quanti­ties of 10% Pd on carbon as catalyst afforded the title compound. For reviews on the Diels–Alder reaction, see: Oppolzer (1991 ▶); Pindur *et al.* (1993 ▶). For a review on asymmetric cyclo­addion processes, see: Pellissier (2012 ▶). For a review on catalytic hydrogenations, see: Brieger & Nestrick (1974 ▶). For a review on asymmetric catalytic hydrogenation processes, see: Knowles (2002 ▶). For discussions on reaction mechanisms with specifics on kinetic and thermodynamic control, see: Lowry & Richardson (1987 ▶); Smith (2012 ▶). For a discussion on Diels–Alder selectivity using maleic anhydride, see: Palmer (2004 ▶).
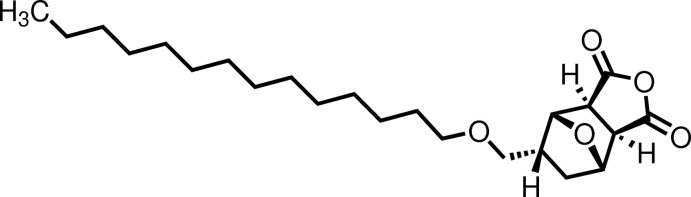


## Experimental

### 

#### Crystal data

C_23_H_38_O_5_
*M* *_r_* = 394.53
Monoclinic, 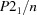

*a* = 6.8541 (5) Å
*b* = 35.206 (4) Å
*c* = 9.2992 (7) Å
β = 99.060 (7)°
*V* = 2216.0 (3) Å^3^
*Z* = 4
Mo *K*α radiation
μ = 0.08 mm^−1^
*T* = 180 K
0.47 × 0.17 × 0.02 mm

#### Data collection

Agilent Xcalibur Eos diffractometer
Absorption correction: multi-scan (*CrysAlis PRO*; Agilent, 2012 ▶) *T*_min_ = 0.865, *T*_max_ = 1.000
9120 measured reflections
4053 independent reflections
2974 reflections with *I* > 2σ(*I*)
*R*_int_ = 0.028

#### Refinement

*R*[*F* ^2^ > 2σ(*F* ^2^)] = 0.049
*wR*(*F* ^2^) = 0.108
*S* = 1.04
4053 reflections
255 parameters
H-atom parameters constrained
Δρ_max_ = 0.18 e Å^−3^
Δρ_min_ = −0.19 e Å^−3^

### 

Data collection: *CrysAlis PRO* (Agilent, 2012 ▶); cell refinement: *CrysAlis PRO*; data reduction: *CrysAlis PRO*; program(s) used to solve structure: *SHELXS97* (Sheldrick, 2008 ▶); program(s) used to refine structure: *SHELXL97* (Sheldrick, 2008 ▶); molecular graphics: *OLEX2* (Dolomanov *et al.*, 2009 ▶); software used to prepare material for publication: *publCIF* (Westrip, 2010 ▶).

## Supplementary Material

Crystal structure: contains datablock(s) I, global. DOI: 10.1107/S1600536812046259/zl2516sup1.cif

Structure factors: contains datablock(s) I. DOI: 10.1107/S1600536812046259/zl2516Isup2.hkl

Supplementary material file. DOI: 10.1107/S1600536812046259/zl2516Isup3.cml

Additional supplementary materials:  crystallographic information; 3D view; checkCIF report

## Figures and Tables

**Table 1:** Hydrogen-bond geometry (Å, °)

*D*—H⋯*A*	*D*—H	H⋯*A*	*D*⋯*A*	*D*—H⋯*A*
C1—H1⋯O1^i^	1.00	2.30	3.163 (2)	144
C4—H4⋯O2^ii^	1.00	2.44	3.377 (2)	156

Symmetry codes: (i) 
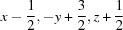
; (ii) 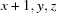.

**Fig. 1.**
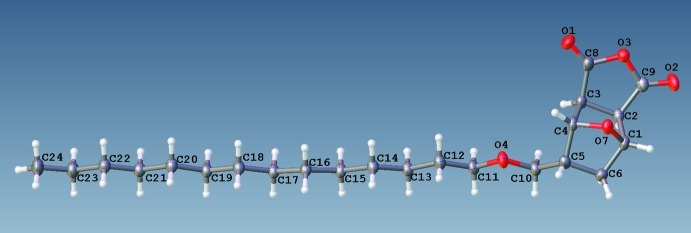
A view of the molecular structure of the title molecule, with the atom numbering. The displacement ellipsoids are drawn at the 50% probability level.

**Fig. 2.**
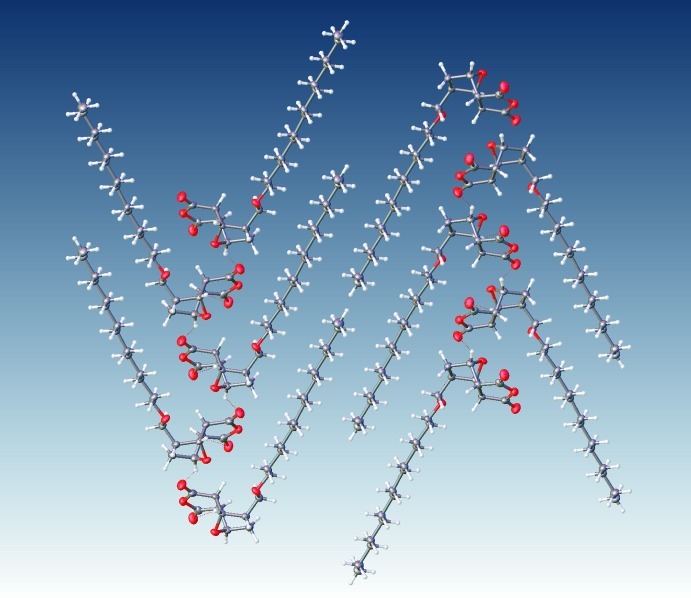
A view along the a-axis of the crystal packing of the title compound, showing the undulating layers that result due to the large polar head groups.

## supplementary crystallographic information

### Comment

The pericyclic [4 + 2] cycloaddition can arguably be considered as one of the most versatile transformations when considering both atom economy and stereochemistry as reported by Oppolzer (1991), Pindur *et al.* (1993), and Pellissier (2012). When coupled to processes which are driven under kinetic or thermodynamic reaction conditions as illustrated by Lowry & Richardson (1987) and Smith (2012), the opportunity to illustrate both modalities on one unified system exists. That is, the irreversible hydrogenation of alkenes as reported by Brieger & Nestrick (1974) and Knowles (2002) and the reversible [4 + 2] cycloaddition when using not cyclopentadiene but furan with maleic anhydride as reported by Palmer (2004) provided us with a platform to illustrate both processes on one system. Upon reversible cycloaddition of a substituted furan with maleic anhydride, the resulting alkene was subjected to catalytic hydrogenation of the alkene.

As the end product was both crystalline and suitable for X-ray analysis, we succeeded in illustrating both reaction pathways of kinetic and thermodynamic driven processes through the establishment of five contiguous stereocenters, as shown in Fig. 1.

The title compound was isolated as the major product in moderate yield and offered definitive evidence of the facial selectivity involved in the catalytic hydrogenation as well as the juxtaposition of the anhydride relative to the bicyclic scaffold as a result of the [4 + 2] cycloaddition. The configurations of the preexisting sites C1, C2, C3, and C4 prior to the hydrogenation of the alkene are *S*, *R*, *S*, and *R* for one of the enantiomers of the racemic mixture, and *R*, *S*, *R*, and *S* for the other, respectively. The configuration of the newly formed stereocenter upon hydrogenation of the chiral racemic mixture is *R* for the former, *S* for the latter, which confirms a profile of kinetic reaction control for the hydrogenation and thermodynamic reaction control for the cycloaddition.

In the solid state structure of the title compound (Fig. 1) the small amount of vibrational motion of the tetradecoxymethyl tail group indicates a significant degree of non-covalent interactions within those domains. No unusual deviations from normal bond distances or bond angles are observed in the title molecule.

In the crystal, molecules are linked head-to-head via C-H···O hydrogen bonds (Table 1) to form V-shaped or folded two-dimensional networks extending in the a and c directions. In the crystal, there are clear hydrophobic and hydrophilic domains (Fig. 2).

### Experimental

The Diels-Alder adduct, 5-tetradecoxymethyl-7-oxabicyclo[2.2.1]hept-5-ene-2,3-dicarboxylic anhydride, was first synthesized by the following method. To a solution of 3-tetradecoxymethylfuran (1.3 g, 4.5 mmol) in toluene (25 ml) was added maleic anhydride (0.56 g, 5.7 mmol). The reaction mixture was allowed to stir at room temperature for a period of 24 h at which time the reaction was determined complete by thin layer chromatography. The reaction mixture was concentrated under reduced pressure and purified by column chromatography (EtOAc/hexanes, 1/4), to afford the *cyclo* adduct. (737 mg, 42% yield). TLC *R_f_* 0.31 (EtOAc/hexanes, 1/4). Spectroscopic data for the Diels-Alder adduct, 5-tetradecoxymethyl-7-oxabicyclo[2.2.1]hept-5-ene-2,3-dicarboxylic anhydride, are available in the archived CIF.

The title compound was prepared by bubbling hydrogen gas into a tetrahydrofuran (50 ml) solution consisting of the Diels-Alder adduct starting material (607 mg, 1.5 mmol) and 10% Pd/C (64 mg) for a period of no less than 90 min. at room temperature. The reaction mixture was then filtered through a plug of Celite and concentrated under reduced pressure. Purification by column chromatography (EtOAc/hexanes, 1/4) afforded the title compound (154 mg, 26% yield). Colourless plate-like crystals were obtained on slow evaporation of a solution in the solvent mixture EtOAc/hexanes (1/4). Spectroscopic data for the title compound are available in the archived CIF.

### Refinement

H atoms were placed in calculated positions and treated as riding atoms: C-H = 0.98, 0.99 and 0.100 Å for CH_3_, CH_2_ and CH H atoms, respectively, with *U*_iso_(H) = k × *U*_eq_(C) where k = 1.5 for CH_3_ H atoms, and = 1.2 for other H atoms.

### Crystal data

**Table:**

C_23_H_38_O_5_	*F*(000) = 864
*M**_r_* = 394.53	*D*_x_ = 1.183 Mg m^−^^3^
Monoclinic, *P*2_1_/*n*	Mo *K*α radiation, λ = 0.7107 Å
Hall symbol: -P 2yn	Cell parameters from 1915 reflections
*a* = 6.8541 (5) Å	θ = 3.2–25.3°
*b* = 35.206 (4) Å	µ = 0.08 mm^−^^1^
*c* = 9.2992 (7) Å	*T* = 180 K
β = 99.060 (7)°	Plate, colourless
*V* = 2216.0 (3) Å^3^	0.47 × 0.17 × 0.02 mm
*Z* = 4	

### Data collection

**Table:**

Agilent Xcalibur Eos diffractometer	4053 independent reflections
Radiation source: Enhance (Mo) X-ray Source	2974 reflections with *I* > 2σ(*I*)
Graphite monochromator	*R*_int_ = 0.028
Detector resolution: 16.0514 pixels mm^-1^	θ_max_ = 25.3°, θ_min_ = 3.2°
ω scans	*h* = −8→8
Absorption correction: multi-scan (*CrysAlis PRO*; Agilent, 2012)	*k* = 0→42
*T*_min_ = 0.865, *T*_max_ = 1.000	*l* = 0→11
9120 measured reflections	

### Refinement

**Table:**

Refinement on *F*^2^	Secondary atom site location: difference Fourier map
Least-squares matrix: full	Hydrogen site location: inferred from neighbouring sites
*R*[*F*^2^ > 2σ(*F*^2^)] = 0.049	H-atom parameters constrained
*wR*(*F*^2^) = 0.108	*w* = 1/[σ^2^(*F*_o_^2^) + (0.0351*P*)^2^ + 0.6309*P*] where *P* = (*F*_o_^2^ + 2*F*_c_^2^)/3
*S* = 1.04	(Δ/σ)_max_ = 0.001
4053 reflections	Δρ_max_ = 0.18 e Å^−^^3^
255 parameters	Δρ_min_ = −0.19 e Å^−^^3^
0 restraints	Extinction correction: *SHELXL*, Fc^*^=kFc[1+0.001xFc^2^λ^3^/sin(2θ)]^-1/4^
Primary atom site location: structure-invariant direct methods	Extinction coefficient: 0.0027 (5)

### Special details

**Table:**

Experimental. Spectroscopic data for the Diels-Alder adduct, 5-tetradecoxymethyl-7-oxabicyclo[2.2.1]hept-5-ene-2,3-dicarboxylic anhydride: ^1^H NMR (300 MHz; CDCl_3_) δ 6.33 (s, 1H), 5.44 (s, 1H), 5.38 (s, 1H), 4.16 (dd, 2H), 3.45 (m, 2H), 3.25 (dd, 2H), 1.56 (m, 2H), 1.27 (b, 22H), 0.90 (t, 3H). Spectroscopic data for the title compound, (1*S**,2*R**,3*S**,4*R**,5*R**)- 5-Tetradecoxymethyl-7-oxabicyclo[2.2.1]heptane-2,3-dicarboxylic anhydride: ^1^H NMR (300 MHz; CDCl_3_) δ 5.00 (dd, 2H), 3.66 (m, 2H), 3.43 (m, 2H), 3.38 (d, 1H), 3.11 (d, 1H), 2.52 (m, 1H), 2.02 (m, 1H), 1.45 (d, 1H), 1.39 (m, 1H), 1.27 (b, 22H), 0.89 (t, 3H).
Geometry. Bond distances, angles etc. have been calculated using the rounded fractional coordinates. All su's are estimated from the variances of the (full) variance-covariance matrix. The cell esds are taken into account in the estimation of distances, angles and torsion angles
Refinement. Refinement of F^2^ against ALL reflections. The weighted R-factor wR and goodness of fit S are based on F^2^, conventional R-factors R are based on F, with F set to zero for negative F^2^. The threshold expression of F^2^ > 2sigma(F^2^) is used only for calculating R-factors(gt) etc. and is not relevant to the choice of reflections for refinement. R-factors based on F^2^ are statistically about twice as large as those based on F, and R- factors based on ALL data will be even larger.

### Fractional atomic coordinates and isotropic or equivalent isotropic displacement parameters (Å^2^)

**Table:**

	*x*	*y*	*z*	*U*_iso_*/*U*_eq_	
O1	−0.1284 (2)	0.69729 (4)	1.26562 (15)	0.0447 (5)	
O2	−0.63572 (18)	0.71813 (4)	1.49758 (15)	0.0411 (5)	
O3	−0.39098 (17)	0.69995 (4)	1.37987 (13)	0.0303 (4)	
O4	0.15463 (17)	0.83205 (4)	1.30869 (13)	0.0313 (4)	
O7	−0.11995 (16)	0.75732 (4)	1.59505 (12)	0.0275 (4)	
C1	−0.3001 (2)	0.77966 (6)	1.57800 (19)	0.0273 (6)	
C2	−0.4085 (2)	0.76561 (6)	1.42974 (18)	0.0253 (6)	
C3	−0.2330 (2)	0.75869 (5)	1.34743 (18)	0.0243 (6)	
C4	−0.0545 (2)	0.77017 (6)	1.46208 (18)	0.0247 (6)	
C5	−0.0436 (2)	0.81327 (6)	1.48478 (18)	0.0271 (6)	
C6	−0.2285 (3)	0.82006 (6)	1.5591 (2)	0.0301 (6)	
C8	−0.2361 (3)	0.71674 (6)	1.32259 (19)	0.0299 (6)	
C9	−0.4970 (2)	0.72724 (6)	1.44200 (19)	0.0283 (6)	
C10	−0.0329 (2)	0.83762 (6)	1.35282 (19)	0.0285 (6)	
C11	0.1794 (2)	0.85616 (6)	1.18939 (18)	0.0273 (6)	
C12	0.3880 (2)	0.85308 (6)	1.15780 (19)	0.0292 (6)	
C13	0.4198 (2)	0.87759 (6)	1.02767 (18)	0.0269 (6)	
C14	0.6329 (2)	0.87751 (6)	0.99874 (18)	0.0267 (6)	
C15	0.6614 (2)	0.89951 (6)	0.86264 (19)	0.0277 (6)	
C16	0.8754 (2)	0.90104 (6)	0.83624 (18)	0.0254 (6)	
C17	0.9015 (2)	0.92066 (6)	0.69409 (19)	0.0274 (6)	
C18	1.1123 (2)	0.91929 (6)	0.66120 (19)	0.0284 (6)	
C19	1.1398 (2)	0.94060 (6)	0.52315 (19)	0.0278 (6)	
C20	1.3486 (2)	0.93808 (6)	0.48634 (19)	0.0295 (6)	
C21	1.3765 (2)	0.96049 (6)	0.35073 (19)	0.0291 (6)	
C22	1.5860 (3)	0.95956 (6)	0.31546 (19)	0.0299 (6)	
C23	1.6097 (3)	0.98168 (6)	0.1793 (2)	0.0353 (7)	
C24	1.8192 (3)	0.98184 (7)	0.1442 (2)	0.0438 (8)	
H1	−0.37790	0.77670	1.65970	0.0330*	
H2	−0.50540	0.78450	1.38020	0.0300*	
H3	−0.24250	0.77370	1.25520	0.0290*	
H4	0.07310	0.75860	1.44520	0.0300*	
H5	0.07600	0.81880	1.55820	0.0320*	
H6A	−0.19290	0.83300	1.65420	0.0360*	
H6B	−0.32960	0.83530	1.49630	0.0360*	
H10A	−0.13950	0.83040	1.27280	0.0340*	
H10B	−0.05000	0.86470	1.37680	0.0340*	
H11A	0.15120	0.88280	1.21340	0.0330*	
H11B	0.08530	0.84870	1.10210	0.0330*	
H12A	0.41690	0.82620	1.13750	0.0350*	
H12B	0.48120	0.86120	1.24470	0.0350*	
H13A	0.33260	0.86820	0.93970	0.0320*	
H13B	0.38050	0.90400	1.04530	0.0320*	
H14A	0.71880	0.88870	1.08380	0.0320*	
H14B	0.67550	0.85090	0.98860	0.0320*	
H15A	0.61300	0.92580	0.87100	0.0330*	
H15B	0.57970	0.88760	0.77720	0.0330*	
H16A	0.92690	0.87480	0.83520	0.0300*	
H16B	0.95530	0.91460	0.91830	0.0300*	
H17A	0.81270	0.90850	0.61290	0.0330*	
H17B	0.86070	0.94750	0.69870	0.0330*	
H18A	1.15080	0.89240	0.65140	0.0340*	
H18B	1.20220	0.93030	0.74460	0.0340*	
H19A	1.10620	0.96770	0.53460	0.0330*	
H19B	1.04600	0.93030	0.44050	0.0330*	
H20A	1.44290	0.94770	0.57010	0.0350*	
H20B	1.38060	0.91110	0.47170	0.0350*	
H21A	1.28550	0.95020	0.26650	0.0350*	
H21B	1.33900	0.98730	0.36400	0.0350*	
H22A	1.67730	0.97010	0.39900	0.0360*	
H22B	1.62450	0.93280	0.30280	0.0360*	
H23A	1.56790	1.00830	0.19130	0.0420*	
H23B	1.52030	0.97070	0.09570	0.0420*	
H24A	1.90850	0.99340	0.22500	0.0660*	
H24B	1.82270	0.99650	0.05510	0.0660*	
H24C	1.86120	0.95570	0.12970	0.0660*	

### Atomic displacement parameters (Å^2^)

**Table:**

	*U*^11^	*U*^22^	*U*^33^	*U*^12^	*U*^13^	*U*^23^
O1	0.0525 (9)	0.0385 (10)	0.0500 (9)	−0.0013 (8)	0.0294 (7)	−0.0091 (8)
O2	0.0253 (7)	0.0399 (10)	0.0616 (9)	−0.0051 (7)	0.0178 (7)	0.0052 (8)
O3	0.0304 (7)	0.0286 (8)	0.0335 (7)	−0.0033 (6)	0.0097 (5)	0.0003 (6)
O4	0.0277 (7)	0.0363 (9)	0.0318 (7)	−0.0014 (6)	0.0104 (5)	0.0123 (6)
O7	0.0271 (6)	0.0345 (9)	0.0217 (6)	−0.0018 (6)	0.0062 (5)	0.0064 (6)
C1	0.0258 (9)	0.0330 (12)	0.0256 (9)	−0.0003 (9)	0.0119 (7)	0.0014 (8)
C2	0.0220 (9)	0.0274 (12)	0.0270 (9)	0.0014 (8)	0.0051 (7)	0.0046 (8)
C3	0.0270 (9)	0.0273 (12)	0.0195 (9)	−0.0010 (8)	0.0066 (7)	0.0050 (8)
C4	0.0220 (9)	0.0303 (12)	0.0237 (9)	−0.0002 (8)	0.0093 (7)	0.0062 (8)
C5	0.0260 (9)	0.0314 (12)	0.0240 (9)	−0.0058 (8)	0.0045 (7)	0.0004 (8)
C6	0.0350 (11)	0.0292 (12)	0.0283 (10)	−0.0026 (9)	0.0114 (8)	−0.0033 (9)
C8	0.0314 (10)	0.0360 (13)	0.0237 (9)	−0.0039 (9)	0.0085 (8)	0.0001 (9)
C9	0.0209 (9)	0.0332 (13)	0.0299 (10)	0.0017 (8)	0.0016 (8)	0.0051 (9)
C10	0.0264 (10)	0.0276 (12)	0.0329 (10)	−0.0032 (8)	0.0095 (8)	0.0046 (9)
C11	0.0295 (10)	0.0277 (12)	0.0258 (9)	−0.0041 (8)	0.0076 (7)	0.0071 (8)
C12	0.0264 (10)	0.0329 (13)	0.0290 (10)	−0.0024 (9)	0.0069 (8)	0.0073 (9)
C13	0.0242 (9)	0.0281 (12)	0.0292 (10)	0.0000 (8)	0.0065 (8)	0.0055 (8)
C14	0.0247 (9)	0.0277 (12)	0.0286 (10)	0.0009 (8)	0.0068 (7)	0.0066 (8)
C15	0.0256 (10)	0.0291 (12)	0.0299 (10)	0.0017 (8)	0.0088 (8)	0.0060 (9)
C16	0.0253 (9)	0.0251 (12)	0.0268 (9)	0.0002 (8)	0.0076 (7)	0.0032 (8)
C17	0.0263 (10)	0.0272 (12)	0.0301 (10)	0.0009 (8)	0.0088 (8)	0.0060 (8)
C18	0.0272 (10)	0.0279 (12)	0.0314 (10)	0.0006 (8)	0.0088 (8)	0.0061 (9)
C19	0.0261 (9)	0.0281 (12)	0.0309 (10)	−0.0007 (8)	0.0094 (8)	0.0038 (9)
C20	0.0284 (10)	0.0324 (12)	0.0293 (10)	0.0011 (9)	0.0091 (8)	0.0054 (9)
C21	0.0296 (10)	0.0306 (12)	0.0284 (10)	−0.0003 (9)	0.0087 (8)	0.0041 (9)
C22	0.0300 (10)	0.0326 (13)	0.0287 (10)	−0.0024 (9)	0.0092 (8)	0.0009 (9)
C23	0.0367 (11)	0.0404 (14)	0.0309 (10)	−0.0048 (10)	0.0120 (8)	0.0042 (10)
C24	0.0439 (12)	0.0493 (16)	0.0428 (12)	−0.0091 (11)	0.0212 (10)	0.0015 (11)

### Geometric parameters (Å, º)

**Table:**

O1—C8	1.191 (2)	C5—H5	1.0000
O2—C9	1.195 (2)	C6—H6A	0.9900
O3—C8	1.392 (2)	C6—H6B	0.9900
O3—C9	1.385 (2)	C10—H10A	0.9900
O4—C10	1.4231 (19)	C10—H10B	0.9900
O4—C11	1.428 (2)	C11—H11A	0.9900
O7—C1	1.452 (2)	C11—H11B	0.9900
O7—C4	1.452 (2)	C12—H12A	0.9900
C1—C2	1.541 (2)	C12—H12B	0.9900
C1—C6	1.524 (3)	C13—H13A	0.9900
C2—C3	1.543 (2)	C13—H13B	0.9900
C2—C9	1.493 (3)	C14—H14A	0.9900
C3—C4	1.545 (2)	C14—H14B	0.9900
C3—C8	1.495 (3)	C15—H15A	0.9900
C4—C5	1.532 (3)	C15—H15B	0.9900
C5—C6	1.555 (3)	C16—H16A	0.9900
C5—C10	1.508 (3)	C16—H16B	0.9900
C11—C12	1.508 (2)	C17—H17A	0.9900
C12—C13	1.530 (3)	C17—H17B	0.9900
C13—C14	1.526 (2)	C18—H18A	0.9900
C14—C15	1.522 (3)	C18—H18B	0.9900
C15—C16	1.526 (2)	C19—H19A	0.9900
C16—C17	1.527 (3)	C19—H19B	0.9900
C17—C18	1.524 (2)	C20—H20A	0.9900
C18—C19	1.524 (3)	C20—H20B	0.9900
C19—C20	1.526 (2)	C21—H21A	0.9900
C20—C21	1.525 (3)	C21—H21B	0.9900
C21—C22	1.523 (3)	C22—H22A	0.9900
C22—C23	1.517 (3)	C22—H22B	0.9900
C23—C24	1.522 (3)	C23—H23A	0.9900
C1—H1	1.0000	C23—H23B	0.9900
C2—H2	1.0000	C24—H24A	0.9800
C3—H3	1.0000	C24—H24B	0.9800
C4—H4	1.0000	C24—H24C	0.9800
			
C8—O3—C9	110.30 (15)	H11A—C11—H11B	108.00
C10—O4—C11	111.43 (13)	C11—C12—H12A	109.00
C1—O7—C4	96.30 (12)	C11—C12—H12B	109.00
O7—C1—C2	101.88 (14)	C13—C12—H12A	109.00
O7—C1—C6	103.56 (13)	C13—C12—H12B	109.00
C2—C1—C6	108.37 (15)	H12A—C12—H12B	108.00
C1—C2—C3	101.00 (12)	C12—C13—H13A	109.00
C1—C2—C9	111.51 (15)	C12—C13—H13B	109.00
C3—C2—C9	104.65 (15)	C14—C13—H13A	109.00
C2—C3—C4	102.17 (13)	C14—C13—H13B	109.00
C2—C3—C8	103.85 (14)	H13A—C13—H13B	108.00
C4—C3—C8	110.84 (15)	C13—C14—H14A	109.00
O7—C4—C3	100.89 (12)	C13—C14—H14B	109.00
O7—C4—C5	101.89 (13)	C15—C14—H14A	109.00
C3—C4—C5	111.74 (14)	C15—C14—H14B	109.00
C4—C5—C6	100.84 (14)	H14A—C14—H14B	108.00
C4—C5—C10	117.28 (15)	C14—C15—H15A	109.00
C6—C5—C10	114.95 (15)	C14—C15—H15B	109.00
C1—C6—C5	101.99 (16)	C16—C15—H15A	109.00
O1—C8—O3	119.31 (18)	C16—C15—H15B	109.00
O1—C8—C3	129.97 (18)	H15A—C15—H15B	108.00
O3—C8—C3	110.71 (15)	C15—C16—H16A	109.00
O2—C9—O3	119.98 (18)	C15—C16—H16B	109.00
O2—C9—C2	129.53 (17)	C17—C16—H16A	109.00
O3—C9—C2	110.48 (13)	C17—C16—H16B	109.00
O4—C10—C5	108.55 (14)	H16A—C16—H16B	108.00
O4—C11—C12	109.93 (14)	C16—C17—H17A	109.00
C11—C12—C13	111.78 (14)	C16—C17—H17B	109.00
C12—C13—C14	113.45 (14)	C18—C17—H17A	109.00
C13—C14—C15	113.41 (14)	C18—C17—H17B	109.00
C14—C15—C16	113.84 (14)	H17A—C17—H17B	108.00
C15—C16—C17	113.70 (13)	C17—C18—H18A	109.00
C16—C17—C18	113.80 (14)	C17—C18—H18B	109.00
C17—C18—C19	113.60 (14)	C19—C18—H18A	109.00
C18—C19—C20	113.90 (14)	C19—C18—H18B	109.00
C19—C20—C21	113.56 (14)	H18A—C18—H18B	108.00
C20—C21—C22	114.35 (14)	C18—C19—H19A	109.00
C21—C22—C23	113.41 (16)	C18—C19—H19B	109.00
C22—C23—C24	114.18 (17)	C20—C19—H19A	109.00
O7—C1—H1	114.00	C20—C19—H19B	109.00
C2—C1—H1	114.00	H19A—C19—H19B	108.00
C6—C1—H1	114.00	C19—C20—H20A	109.00
C1—C2—H2	113.00	C19—C20—H20B	109.00
C3—C2—H2	113.00	C21—C20—H20A	109.00
C9—C2—H2	113.00	C21—C20—H20B	109.00
C2—C3—H3	113.00	H20A—C20—H20B	108.00
C4—C3—H3	113.00	C20—C21—H21A	109.00
C8—C3—H3	113.00	C20—C21—H21B	109.00
O7—C4—H4	114.00	C22—C21—H21A	109.00
C3—C4—H4	114.00	C22—C21—H21B	109.00
C5—C4—H4	114.00	H21A—C21—H21B	108.00
C4—C5—H5	108.00	C21—C22—H22A	109.00
C6—C5—H5	108.00	C21—C22—H22B	109.00
C10—C5—H5	108.00	C23—C22—H22A	109.00
C1—C6—H6A	111.00	C23—C22—H22B	109.00
C1—C6—H6B	111.00	H22A—C22—H22B	108.00
C5—C6—H6A	111.00	C22—C23—H23A	109.00
C5—C6—H6B	111.00	C22—C23—H23B	109.00
H6A—C6—H6B	109.00	C24—C23—H23A	109.00
O4—C10—H10A	110.00	C24—C23—H23B	109.00
O4—C10—H10B	110.00	H23A—C23—H23B	108.00
C5—C10—H10A	110.00	C23—C24—H24A	109.00
C5—C10—H10B	110.00	C23—C24—H24B	109.00
H10A—C10—H10B	108.00	C23—C24—H24C	109.00
O4—C11—H11A	110.00	H24A—C24—H24B	110.00
O4—C11—H11B	110.00	H24A—C24—H24C	110.00
C12—C11—H11A	110.00	H24B—C24—H24C	109.00
C12—C11—H11B	110.00		
			
C9—O3—C8—O1	179.49 (16)	C8—C3—C4—O7	−74.70 (16)
C9—O3—C8—C3	0.42 (19)	C8—C3—C4—C5	177.69 (13)
C8—O3—C9—O2	−179.69 (16)	C2—C3—C8—O1	−178.60 (19)
C8—O3—C9—C2	−1.05 (18)	C2—C3—C8—O3	0.34 (18)
C11—O4—C10—C5	−176.33 (15)	C4—C3—C8—O1	−69.6 (2)
C10—O4—C11—C12	172.98 (15)	C4—C3—C8—O3	109.38 (15)
C4—O7—C1—C2	58.09 (15)	O7—C4—C5—C6	−38.51 (14)
C4—O7—C1—C6	−54.37 (15)	O7—C4—C5—C10	−164.10 (12)
C1—O7—C4—C3	−57.67 (15)	C3—C4—C5—C6	68.45 (16)
C1—O7—C4—C5	57.54 (13)	C3—C4—C5—C10	−57.14 (17)
O7—C1—C2—C3	−35.03 (17)	C4—C5—C6—C1	4.96 (16)
O7—C1—C2—C9	75.68 (15)	C10—C5—C6—C1	132.10 (16)
C6—C1—C2—C3	73.78 (17)	C4—C5—C10—O4	−69.48 (17)
C6—C1—C2—C9	−175.52 (14)	C6—C5—C10—O4	172.27 (15)
O7—C1—C6—C5	30.12 (16)	O4—C11—C12—C13	178.34 (15)
C2—C1—C6—C5	−77.52 (15)	C11—C12—C13—C14	175.87 (16)
C1—C2—C3—C4	−0.33 (18)	C12—C13—C14—C15	175.72 (16)
C1—C2—C3—C8	115.01 (16)	C13—C14—C15—C16	177.53 (16)
C9—C2—C3—C4	−116.23 (15)	C14—C15—C16—C17	175.87 (17)
C9—C2—C3—C8	−0.89 (17)	C15—C16—C17—C18	−175.12 (17)
C1—C2—C9—O2	71.3 (2)	C16—C17—C18—C19	−177.12 (16)
C1—C2—C9—O3	−107.15 (15)	C17—C18—C19—C20	−177.78 (16)
C3—C2—C9—O2	179.68 (18)	C18—C19—C20—C21	−178.20 (16)
C3—C2—C9—O3	1.21 (17)	C19—C20—C21—C22	177.79 (16)
C2—C3—C4—O7	35.43 (17)	C20—C21—C22—C23	179.38 (17)
C2—C3—C4—C5	−72.18 (16)	C21—C22—C23—C24	178.66 (17)

### Hydrogen-bond geometry (Å, º)

**Table:**

*D*—H···*A*	*D*—H	H···*A*	*D*···*A*	*D*—H···*A*
C1—H1···O1^i^	1.00	2.30	3.163 (2)	144
C4—H4···O2^ii^	1.00	2.44	3.377 (2)	156

Symmetry codes: (i) *x*−1/2, −*y*+3/2, *z*+1/2; (ii) *x*+1, *y*, *z*.

## References

[bb1] Agilent (2012). *CrysAlis PRO* Agilent Technologies, Yarnton, England.

[bb2] Brieger, G. & Nestrick, T. J. (1974). *Chem. Rev.* **74**, 567–580.

[bb3] Dolomanov, O. V., Bourhis, L. J., Gildea, R. J., Howard, J. A. K. & Puschmann, H. (2009). *J. Appl. Cryst.* **42**, 339–341.

[bb4] Knowles, W. S. (2002). *Angew. Chem. Int. Ed.* **41**, 1998–2007.

[bb5] Lowry, T. H. & Richardson, K. S. (1987). *Mechanism and Theory in Organic Chemistry*, 3rd ed. New York: Harper & Row.

[bb6] Oppolzer, W. (1991). *Comprehensive Organic Synthesis*, edited by B. M. Trost & I Fleming. Oxford: Pergamon.

[bb7] Palmer, D. R. J. (2004). *J. Chem. Educ.* **81**, 1633–1635.

[bb8] Pellissier, H. (2012). *Tetrahedron*, **68**, 2197–2232.

[bb9] Pindur, U., Lutz, G. & Otto, C. (1993). *Chem. Rev.* **93**, 741–761.

[bb10] Sheldrick, G. M. (2008). *Acta Cryst.* A**64**, 112–122. 10.1107/S0108767307043930 18156677

[bb11] Smith, M. B. (2012). *March’s Advanced Organic Chemistry: Reactions, Mechanisms, and Structure*, 7th ed. New York: Wiley.

[bb12] Westrip, S. P. (2010). *J. Appl. Cryst.* **43**, 920–925.

